# Gli1 contributes to cellular resistance to cisplatin through altered cellular accumulation of the drug

**DOI:** 10.3892/or.2014.3257

**Published:** 2014-06-12

**Authors:** LAUREN AMABLE, JASON FAIN, ELAINE GAVIN, EDDIE REED

**Affiliations:** 1 National Institute on Minority Health and Health Disparities, National Institutes of Health, Bethesda, MD 20892, USA; 2 Mitchell Cancer Institute, University of South Alabama, Mobile, AL 36604, USA

**Keywords:** cisplatin resistance, Gli1, cisplatin transport, ovarian cancer, DNA repair, DNA adduct

## Abstract

Cellular resistance to platinum anticancer compounds is governed by no less than two molecular processes; DNA repair and cellular accumulation of drug. Gli1 is an upstream regulator of nucleotide excision repair, effecting this process through c-jun. We, therefore, investigated whether Gli1 plays a role in cellular accumulation of cisplatin. Using a Gli1-specific shRNA, we explored the role of Gli1 in the cellular accumulation and efflux of cisplatin, in cisplatin-resistant A2780-CP70 human ovarian cancer cells. When Gli1 is inhibited, cellular uptake of cisplatin was approximately 33% of the level of uptake under control conditions. When Gli1 is inhibited, cellular efflux of cisplatin was completely abrogated, over a 12-h period of observation. We assayed nuclear lysates from these cells, for the ability to bind the DNA sequence that is the Gli-binding site (GBS) in the 5′UTR for each of five known cisplatin transmembrane transporters. Four of these transporters are active in cisplatin uptake; and, one is active in cisplatin efflux. In each case, nuclear lysate from A2780-CP70 cells binds the GBS of the respective cisplatin transport gene. We conclude that Gli1 plays a strong role in total cellular accumulation of cisplatin in these cells; and, that the combined effects on cellular accumulation of drug and on DNA repair may indicate a role for Gli1 in protecting cellular DNA from lethal types of DNA damage.

## Introduction

Cancer stem cells have the phenotype of a high level of resistance to a range of anticancer agents, including platinum compounds. Maintenance of cancer stem cells is dependent on the Hedgehog (Hh) pathway. The Hh role in platinum resistance is related in part to the positive transcription factor Gli1 and its role in the regulation of c-jun and ERCC1 (1,2). The knockdown of Gli1 using shRNA results in a series of events: the c-jun phosphorylation pattern changes from the anti-apoptotic Ser63/73 to the pro-apoptotic Thr91/93; c-jun-dependent DNA repair genes are not upregulated; cisplatin-DNA adduct repair is markedly reduced; and cells are sensitized to cisplatin by a factor of six (1).

Cellular resistance to cisplatin is multifactorial (3–5). One of the factors that controls the level of cisplatin resistance is altered cellular accumulation of drug (4–7). Cellular accumulation of cisplatin is regulated by several processes, including passive diffusion, carrier-mediated uptake and carrier-mediated efflux. In the excellent review by Hall *et al* (8), the five known mechanisms of cisplatin uptake and the four known mechanisms of cisplatin efflux are summarized. Uptake of cisplatin is mediated by: i) passive diffusion; ii) a protein gate, linked to the Na^+^ K^+^ -ATPase pump; iii) fluid phase endocytosis; iv) organic cation transporter (OCT) 1–3 proteins; and v) copper transporter CTR1 and CTR2 proteins. Efflux is mediated by: i) melanosomes; ii) ATP7B dependent vesicles; iii) ATP7A protein; and iv) MRP1–5 proteins.

Uptake of cisplatin is mediated by proteins that have multiple transport functions. Specifically OCT1, OCT2 and OCT3 function as transporters of organic cations, monoamine neurotransmitters, xenobiotics and various drugs. OCT1, OCT2 and OCT3 transport cisplatin and oxaliplatin. Uptake of cisplatin is also mediated by two copper transporters: CTR1 and CTR2. CTR1 and CTR2 normally function for copper influx. Both of these CTR transporters have been shown to transport cisplatin and carboplatin, but CTR1 is additionally involved in the transport of oxaliplatin.

Efflux of cisplatin occurs through the ATPase copper transporters ATP7A and ATP7B. The ATP7A and ATP7B transporters are both involved in copper sequestration and efflux and transport both cisplatin and carboplatin.

In our examination of the molecular sequences of the genes discussed above, we found potential Gli-binding sites (GBS) in five of these genes: OCT1, OCT2, OCT3, CTR1 and ATP7B. These potential Gli binding sites are given in Fig. 2A. In the present study, we explore whether inhibition of Gli1 has any effect on total cellular accumulation of cisplatin in A2780-CP70 cisplatin-resistant human ovarian cancer cells. We also examined whether there is any evidence that the Gli- binding sites for these genes, would be recognized by nuclear lysates from this cell line. Our results suggest that Gli1 may play a strong role in modulating total cellular accumulation of cisplatin in these cells, through altered drug uptake and altered drug efflux.

## Materials and methods

### Cells

The cisplatin resistant A2780-CP70 ovarian cancer cells were used in all experiments. Cells were retrieved from a frozen stock and experiments were performed between passages 5 and 30. Cells were cultured in RPMI-1640 media (Gibco/Invitrogen, Carlsbad, CA, USA) supplemented with 10% fetal bovine serum (Gibco), L-glutamine (Gibco), insulin (Sigma-Aldrich, St. Louis, MO, USA) and penicillin/streptomycin (Gibco). During active experiments, cells were carried in media without penicillin/streptomycin.

### Whole cell platinum analysis

Cells were treated under two experimental conditions. One set of cells were treated with anti-Gli1 shRNA at an IC_20_ dose for 24 h, prior to treatment with cisplatin. Gli1 was targeted for degradation using shRNA specific for Gli1 (1). Control cells were treated with scrambled shRNA at the same micromolar dose for 24 h, prior to cisplatin treatment.

A2780-CP70 cells were seeded at 2×10^6^ in a 10-cm^2^ dish. The following morning, cells were transfected with Gli1 or scrambled shRNA using Lipofectamine according to the manufacturer’s instructions (Invitrogen). Twenty-four hours later cells were treated with 30 μM cisplatin for 1 h, the IC_50_ dose when cisplatin is used alone. Cisplatin-containing media was then removed and plates were washed with cold PBS. The zero hour time point was immediately after the 1-h cisplatin dose. Cells were harvested by trypsinization and collected.

For the 12-h time-point, cisplatin-containing media was removed after the 1-h drug exposure. Cells were washed with PBS and fresh cisplatin-free media was then added. Cells were collected by trypsinization 12 h later. Cells were harvested by centrifugation and counted by trypan blue dye exclusion assays. The collected cell pellets were stored at −80°C until analysis.

Cell pellets were prepared for atomic absorption spectroscopy (AAS) analysis by wet ashing as previously described (9). Briefly, cell pellets were treated with 0.5 ml nitric acid and incubated in a water bath at 100°C for 5 min. Polypropylene 15 ml conical tubes were used. Samples were cooled to room temperature under running water. Hydrogen peroxide, 0.5 ml of 30%, was added to the tube and re-submerged into the water bath at 100°C for 5 min. Samples were again cooled to room temperature before analysis by AAS.

Platinum in each sample was measured by AAS using a Perkin Elmer 600 AAnalyst AAS machine with Zeeman background correction and a platinum lamp (Perkin-Elmer, Walthan, MA, USA). Platinum standards were used to generate a standard curve. Total platinum per 1×10^6^ cells was determined based on the cell counts and the total platinum in each sample. Total cellular platinum per million cells at 0 h, was compared with the total platinum level per million cells at 12 h.

### Binding site search

Genomic sequences containing 10 kB upstream of the translation start site were obtained from Ensembl (www.ensembl.org) for the following transporter genes: ATP7A (ATP7A-001), ATP7B (ATPB-001), CTR1 (SLC31A1-001), CTR2 (SLC31A2-001), OCT1 (SLC22A1-001), OCT2 (SLC22A2-001) and OCT3 (SLC22A3-001). The following known Gli-binding sites were used in the search parameters: GAGCAGCCA, GACGACCCC, GGCCCCCCA, GACCGCCCC, AACCAACCCC, GTCCTCCCA, GACCAC CCA (10), GAACACCCA, CACCACCCA and GACCACCAA (11). A pairwise BLAST (basic local alignment search tool, bl2seq, http://blast.ncbi.nlm.nih.gov/Blast.cgi) was performed to compare the promoter region with each GBS. The parameters of the pairwise BLAST were kept at the default setting except for the word size was changed from 11 to 7. Activator protein 1 (AP1) binding sites were searched in each transporter promoter using the following sequence TGAGTCA (12,13) and keeping in the same pairwise BLAST search parameters.

### Electrophoretic mobility shift assays (EMSAs)

The putative Gli-binding sites found in the promoters in the listed transporters were synthesized as DNA oligonucleotides and the reverse compliment containing a 5′ biotin were obtained for the following transporters: ATP7A (AATCGTAT**GAACACC CA**TATACCCA), CTR2 (AAATAAAT**TGGGTGGTC**AGA GTGGC), OCT1 (TCAGCCCT**TGGGTGGTC**GATGGGAC, GTCCATGC**TTGGTGGTC**TTTTACCA), OCT2 (TAAGTTC C**TGGCTGCTC**GGGGCACT) and OCT3 (AACCGCAA**G TCCTCCCA**AGGCCTTG, TCTGGGGG**TGGGTGGTGG** TTTTATC) (Integrated DNA Technologies, Coralville, IA, USA). Each oligonucleotide was resuspended in Milli-Q-water at a final concentration of 100 μM. Double stranded DNA (dsDNA) probes were generated by adding 1 μM of the forward and reverse compliment oligonucleotides in annealing buffer (10 mM Tris, 1 mM EDTA, 50 mM NaCl, pH 8.0) and heated to 95°C and cooled to room temperature at a rate of 1°C/min.

Nuclear lysate was prepared from A2780-CP70 as previously described (1). Protein concentration was determined using BCA kit (Thermo Pierce, Rockford, IL, USA). The EMSA DNA-binding reaction was carried out using LightShift Chemiluminescent EMSA kit (Thermo Pierce). Each reaction consisted of 20 fmol of biotin labeled dsDNA, 1 μg poly (dI-dC), 20 μg nuclear lysate in a 20 μl of reaction buffer (40 mM HEPES, 25 mM KCl, 10 mM MgCl_2_, 10 mM ZnSO_4_, 500 μM EDTA, 10% glycerol, pH 7.8). The DNA-binding reaction was incubated on ice for 30 min and 5 μl of loading buffer was added. Samples were separated on a 6% native polyacrylamide gel, and transferred to a positively charged PVDF membrane (BrightStar Plus; Ambion, Austin, TX, USA). The dsDNA biotin probe was visualized using the reagents of the kit.

## Results

### Anti-Gli1 shRNA reduces cisplatin influx and shuts down cisplatin efflux

**Figure 1:**
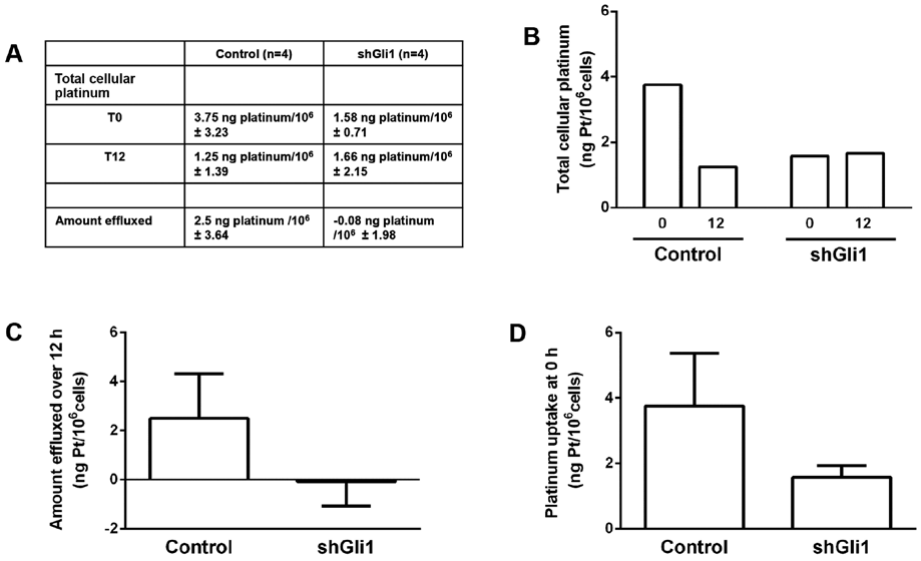
Cells treated with anti-Gli1 shRNA have reduced uptake and efflux of cisplatin, in comparison to control cells. A2780-CP70 cells were transfected with Gli1 or scrambled control shRNA for 24 h. Cells were treated for 1 h with an IC_50_ dose of cisplatin, 30 μM. Time zero began immediately after the 1-h cisplatin treatment to measure uptake. Cells were allowed to recover for 12 h to measure efflux. Cells were collected and whole cell platinum was measured by AAS. (A) Table of numerical results of the data, including means and standard deviations. (B) Total cellular platinum levels at time 0 and after the 12-h recovery period. (C) Cellular platinum efflux after 12 h. (D) Cellular platinum uptake results at time zero.

We first assessed total cellular platinum in these cells, after pretreatment with anti-Gli1 shRNA for 24 h, followed by cisplatin treatment for 1 h. This was compared to pretreatment for 24 h with scrambled shRNA control followed by cisplatin treatment for 1 h, at the same cisplatin dose. As shown in Fig. 1A and B, under control conditions, cells accumulated 3.75 ng Pt/10^6^ cells, after a 1 h cisplatin exposure. Total cellular platinum was also measured 12 h after cisplatin was removed from cells. Total cellular platinum was reduced to 1.25 ng Pt/10^6^ cells, indicating that these cells effluxed 2.5 ng Pt/10^6^ cells over this 12-h period. When cells were treated with anti-Gli1 shRNA, the platinum level at 0 h was 1.58 ng Pt/10^6^ cells, which was essentially unchanged 12 h later, measured at 1.66 ng Pt/10^6^ cells. This indicates that under control conditions, 67% of total cellular platinum was removed over 12 h (3.75 down to 1.25 ng). Furthermore, when cells were pretreated with anti-Gli1 shRNA, these cells were unable to efflux platinum over the same 12-h period. Fig. 1C graphically shows the differences with respect to total drug effluxed during the 12-h experiment.

Fig. 1D, graphically shows the differences between conditions with respect to the initial levels of total cellular platinum. When cells were pretreated with scrambled shRNA total cellular drug was 3.75 vs. 1.58 units when pretreated with anti-Gli1 shRNA; a 58% reduction in drug uptake. This suggests that drug uptake was greatly reduced by the inhibition of Gli1.

### Nuclear lysate recognizes the GBS of each of five platinum transport genes

**Figure 2:**
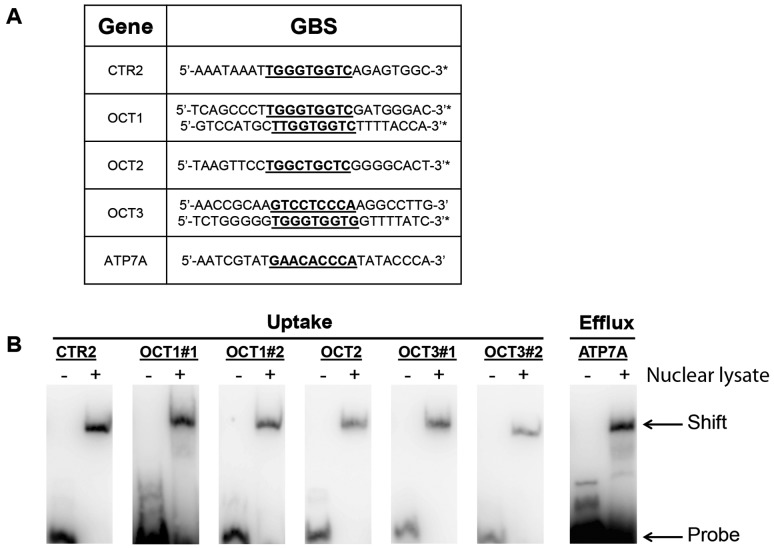
Nuclear lysate from A2780-CP70 cells bind the GBS found in cisplatin transporters. (A) Table displaying the platinum transporters and the GBS sequence, underlined, found in the 5′UTR. A reverse compliment GBS is indicated by an asterisk. In each case the eight base pair sequence that flanks the GBS in that specific gene is listed. This was incorporated in the synthesized probe. (B) EMSAs were performed to assess if a nuclear lysate protein binds the GBS identified in the cisplatin transporters listed in (A). Each GBS was analyzed using a negative control (−), which contains the biotin-labeled oligonucleotide probe and no nuclear lysate. The positive control (+), contains A2780-CP70 nuclear lysate with the biotin-labeled GBS of the respective transporter; showing the resultant shift. All transporter EMSAs exhibited a shift in the positive control suggesting a Gli protein binds the GBS.

We studied seven of 12 known platinum transport genes, for the possibility of harboring one or more Gli-binding sites in their 5′UTR. The genes we studied were CTR1, CTR2, ATP7A, ATP7B, OCT1, OCT2 and OCT3. Approximately 10 kB upstream of each gene was obtained and searched for the binding sites using BLAST (http://blast.ncbi.nlm.nih.gov/Blast.cgi). For two of these seven genes, there was no indication of a GBS in the 5′UTR; CTR1 and ATP7B. For five genes that contained a GBS in the 5′UTR, those sequences are listed in Fig. 2A. Two of these genes, OCT1 and OCT3, have two GBS sites each, which are given in Fig. 2A.

We next assessed nuclear lysates from A2780-CP70 cells, for the ability to bind the synthesized Gli-binding sites for each of the platinum transporters listed in Fig. 2A. In Fig. 2B, we show the EMSAs for each GBS for each gene. The EMSA is run with the synthetic GBS mixed with nuclear lysate. The negative control, GBS alone, is run to check for gross abnormalities of the probe.

CTR2, OCT1, OCT2 and OCT3, are platinum influx proteins/genes. In each case the GBS probe demonstrated the predicted shift within the nuclear lysate lane. ATP7A is a platinum efflux protein/gene. In this case as well, the GBS probe demonstrated the predicted shift. Therefore, for each of the five genes studied, and a total of seven GBS probes, the nuclear lysate of these cells generated the predicted shift. This indicates that a GBS-binding protein exists in the nuclear lysate of these cells for each of these five platinum transport genes. As shown in Fig. 1, when Gli1 is disrupted by the use of anti-Gli1 shRNA, cellular influx of cisplatin is reduced and cellular efflux of cisplatin is abrogated.

### Comparing cellular accumulation and DNA repair in the cells

**Figure 3:**
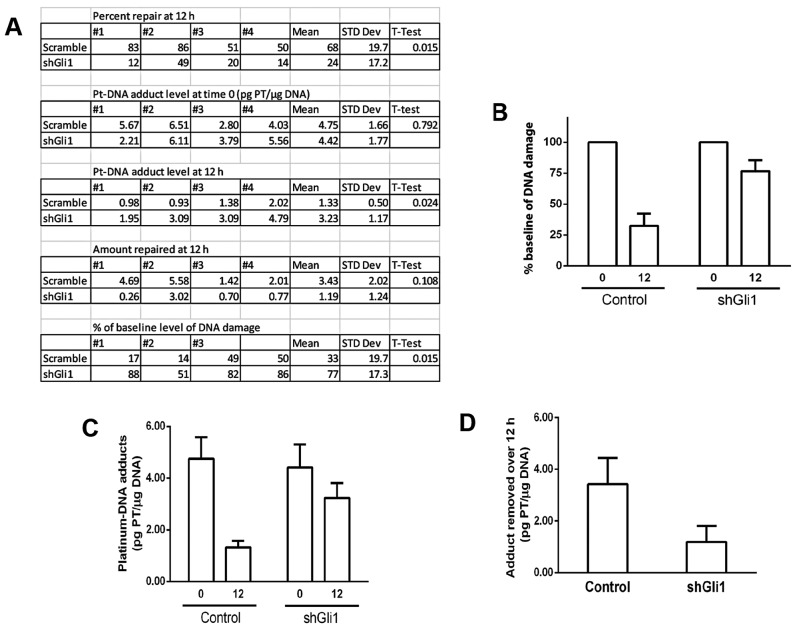
Repair of DNA-platinum adducts is inhibited in cells treated with Gli1 shRNA. A2780-CP70 cells were treated for 24 h with anti-Gli1 or scrambled control shRNA. Cells were then treated with an IC_50_ dose of cisplatin (30 μM) for 1 h. Cells were collected and DNA was isolated to measure DNA-platinum adducts at time 0 (immediately after cisplatin treatment) and 12 h later. (A) Table listing all numerical data of DNA-platinum adduct experiments. (B) Graph of percent baseline of DNA damage. (C) Graph of platinum-DNA adducts at time 0 and 12 h. (D) Graph of platinum-DNA adducts removed over the 12-h period. Cells treated with anti-Gli1 shRNA display reduced levels of DNA repair in comparison to controls.

We have extended the studies we previously performed with respect to the repair of platinum-DNA adducts. This is summarized in Fig. 3. As we have reported (1), the repair of platinum-DNA damage is greatly inhibited by using anti-Gli1 shRNA in these cells. Fig. 3A, gives the raw data from n=4. Fig. 3A–C show that under control conditions, ~68% repair occurs for platinum-DNA damage over 12 h. When cells are treated with anti-Gli1 shRNA, ~24% repair occurs (P=0.015). Fig. 3A and D, show that the absolute amount of DNA adduct repaired over the 12-h time frame, is ~3-fold greater under control conditions, as compared to when Gli1 is inhibited (3.43 vs. 1.19 units).

**Figure 4:**
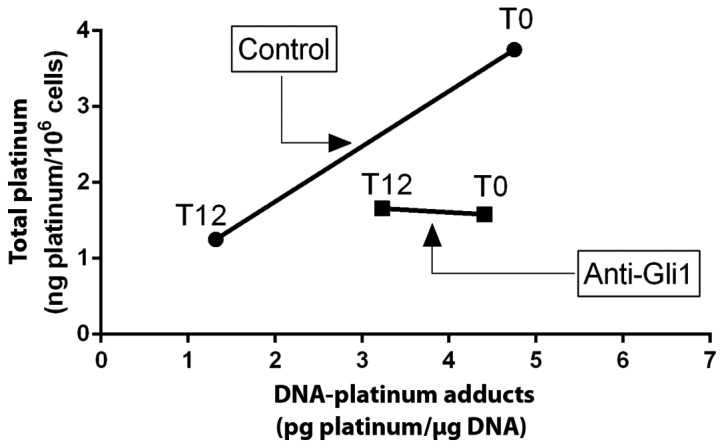
Comparison of DNA damage with whole cell platinum levels in A2780-CP70 cells treated with Gli1 shRNA. Total platinum-DNA damage is plotted against total cellular platinum at time zero (immediately after the 1-h cisplatin exposure) and at 12 h. This is done for shRNA scrambled controls; and for anti-Gli1 shRNA treated cells. Cells treated with Gli1 shRNA display similar levels of DNA-platinum adduct at time zero as do control cells, although drug uptake is reduced. After the 12-h recovery period, Gli1 shRNA treated cells show no change in total cellular platinum and only a 23% repair of the DNA-platinum adducts. Control cells effluxed 67% of the total cellular platinum and repaired 68% of the DNA-platinum adducts. Gli1 is, therefore, involved in three molecular processes: DNA repair, drug accumulation and drug efflux.

We next sought to compare these two experimental conditions, in a manner where total cellular drug and total DNA damage could be assessed concurrently, at 0 h and at 12 h. This is graphically shown in Fig. 4. Here, total cellular platinum is plotted on the vertical axis, and total DNA damage is assessed on the horizontal axis. Cells treated under control conditions are plotted at 0 h and at 12 h. Cells treated with anti-Gli1 shRNA are plotted at 0 h and at 12 h.

Fig. 4 shows is that at time zero (immediately after the 1-h exposure to cisplatin), cells treated with anti-Gli1 shRNA had less than half the total cellular platinum than control cells treated at the same cisplatin dose. This illustrates that inhibition of Gli1, in this manner, greatly inhibits cisplatin uptake in these cells. Also of interest, is that at this IC_50_ cisplatin dose, the total amount of DNA damage experienced by these cells is almost exactly the same, ~4.5 units. This is consistent with previous reports of the relationships between DNA damage levels and cisplatin IC_50_ values in other cell lines (5,14).

Also shown in Fig. 4, under control conditions, reductions in total cellular levels of drug and removal of DNA damage, appear to be phenotypically linked. In contrast, in cells where Gli1 is inhibited, DNA repair continues to a limited degree although cellular efflux of drug is frozen. Previous studies from this group suggest that in these cells, DNA repair and cellular accumulation of drug may be linked processes (5). It appears from Fig. 4, that this linkage can be disrupted through inhibition of Gli1.

## Discussion

Cellular resistance to cisplatin has been associated with the phenotypic linkage of DNA repair and cellular accumulation of drug, since the mid-1980s. Eastman was the first to show in a cellular system, that for levels of cisplatin resistance to ~10-fold over baseline, these two molecular processes go hand-in-hand (15–17). The consensus belief is that this level of resistance was probably the range that was clinically relevant to human patients (18). Parker and colleagues were the first to show that in human ovarian cancer cells, levels of resistance up to ~13-fold over baseline were associated with phenotypic linkage of DNA repair and cellular accumulation of drug (5). This was also observed in human T lymphocyte cell lines (19). Masuda *et al* (20) and Ferry and colleagues (21) confirmed in a series of studies in human ovarian cancer cells, that at this level of resistance, DNA repair was very important. They also showed that at levels of resistance in the range above 30-fold over baseline, cytosolic inactivation of drug appeared to become the predominant molecular mechanism of cisplatin resistance (22,23).

In the present study we report on the A2780-CP70 cell line, where these two processes are linked. Here, we show that Gli1 appears to be an important common molecular entity that controls cellular uptake of drug and cellular efflux of drug. Previously we showed that Gli1 is critical to platinum-DNA repair.

Enhanced DNA repair is associated with cisplatin resistance. The involvement of Gli1 in DNA damage and repair pathways has been reported by several groups (1,24,25). Mazumdar and colleagues demonstrated that inhibition of Gli1 using small molecule inhibitors induces double strand DNA breaks (DSBs) coupled with reduced DNA repair in colon cancer (26,27). Leonard and colleagues demonstrated increased expression of Gli1 in HEK293 cells resulted in the inability to activate Chk1 after ionizing radiation (25).

Our group has identified the importance of Gli1 in the regulation of nucleotide excision repair and base excision repair in response to platinum DNA damage (1). Gli1 shRNA treated cells exhibited a switch in the c-jun phosphorylation cascade, from a pro-survival to a pro-apoptotic pattern. In response to cisplatin treatment, the upregulation of DNA repair genes by c-jun is blocked and cells are unable to repair platinum-DNA lesions.

Our laboratory identified the isoform of Gli1, the 130-kDa isoform, responsible for upregulating c-jun (28). Gli1 has 5 known isoforms: full-length, two mRNA splice variants, and two post-translational truncations of the full-length protein. The 130-kDa Gli1 isoform is generated by an N-terminal truncation of the full-length protein resulting in the removal of the N-terminal Degron domain and the Sufu-binding domain. Ovarian cancer specimens express higher levels of the 130-kDa isoform in comparison to non-cancer specimens. Additionally, the 130-kDa isoform was expressed at higher levels in the cisplatin resistant cell lines A2780-CP70 and A2780-CIS when compared with the parental cell line A2780. This opens the possibility that the Gli1 130-kDa isoform may play a role in both components of cisplatin drug resistance; i.e., DNA repair and cellular accumulation of drug.

There are a large number of genes/proteins associated with platinum transport across cellular membranes. This has been reviewed by Hall *et al* (8). Many of these genes have been shown to be highly conserved, with analogs that exist in grapes (29), rice (30), barley (31) and a range of vertebrates. In humans, these genes tend to be highly expressed in kidney, in the inner ear, and in various tissues throughout the body (8). In the present study, we focused on those transporters where there is a Gli-binding site in the 5′UTR of that specific gene. For these transporters (Fig. 2), there exists the possibility that Gli1 may have direct impact on the regulation of those genes.

We further analyzed the 5′UTR of these genes for binding sites for other regulatory proteins. For the transporters we examined, we could find binding sites for either: Gli, c-jun or AP1 (data not shown). We have previously demonstrated that Gli1 effects changes in cisplatin-DNA adduct repair through its influence on c-jun (1). c-jun heterodimerizes with c-fos to form the transcriptionally active AP1 (32). This suggests that when Gli1 is inhibited by using anti-Gli1 shRNA, there may also be a strong indirect effect on the regulation of some cisplatin transporters, through Gli1′s modulation of c-jun or AP1.
